# Biogenic Sulfur-Based Chalcogenide Nanocrystals: Methods of Fabrication, Mechanistic Aspects, and Bio-Applications

**DOI:** 10.3390/molecules27020458

**Published:** 2022-01-11

**Authors:** Oscar P. Yanchatuña Aguayo, Lynda Mouheb, Katherine Villota Revelo, Paola A. Vásquez-Ucho, Prasad P. Pawar, Ashiqur Rahman, Clayton Jeffryes, Thibault Terencio, Si Amar Dahoumane

**Affiliations:** 1 School of Biological Sciences and Engineering, Yachay Tech University, Hacienda San José s/n, San Miguel de Urcuquí 100119, Ecuador; oscar.yanchatuna@yachaytech.edu.ec (O.P.Y.A.); katherine.villota@yachaytech.edu.ec (K.V.R.); paola.vasquez@yachaytech.edu.ec (P.A.V.-U.); 2 Laboratoire de Recherche de Chimie Appliquée et de Génie Chimique, Hasnaoua I, Université Mouloud Mammeri B.P.17 RP, Tizi-Ouzou 15000, Algeria; lynda.mouheb@ummto.dz; 3 Nanobiomaterials and Bioprocessing Laboratory (NABLAB), Dan F. Smith Department of Chemical Engineering, Lamar University, P.O. Box 10051, Beaumont, TX 77710, USA; ppawar@lamar.edu (P.P.P.); cjeffryes@lamar.edu (C.J.); 4 Center for Midstream Management and Science, Lamar University, 211 Redbird Ln., P.O. Box 10888, Beaumont, TX 77710, USA; arahman2@lamar.edu; 5 Center for Advances in Water and Air Quality, Lamar University, Beaumont, TX 77710, USA; 6 School of Chemical Sciences and Engineering, Yachay Tech University, Hacienda San José s/n, San Miguel de Urcuquí 100119, Ecuador; 7 Department of Chemical Engineering, Polytechnique Montréal, C.P. 6079, Succ. Centre-Ville, Montréal, QC H3C 3A7, Canada

**Keywords:** sulfur-based nanoparticles, quantum dots, biosynthesis, sustainability, properties, bio-applications

## Abstract

Bio-nanotechnology has emerged as an efficient and competitive methodology for the production of added-value nanomaterials (NMs). This review article gathers knowledge gleaned from the literature regarding the biosynthesis of sulfur-based chalcogenide nanoparticles (S-NPs), such as CdS, ZnS and PbS NPs, using various biological resources, namely bacteria, fungi including yeast, algae, plant extracts, single biomolecules, and viruses. In addition, this work sheds light onto the hypothetical mechanistic aspects, and discusses the impact of varying the experimental parameters, such as the employed bio-entity, time, pH, and biomass concentration, on the obtained S-NPs and, consequently, on their properties. Furthermore, various bio-applications of these NMs are described. Finally, key elements regarding the whole process are summed up and some hints are provided to overcome encountered bottlenecks towards the improved and scalable production of biogenic S-NPs.

## 1. Introduction

Nanotechnology deals with the study and engineering of nanoparticles (NPs) or nanomaterials (NMs), which are materials with at least one dimension between 1 nm to 100 nm [1]. Over the last decades, this discipline has grown and witnessed formidable developments due to the sustained growth and diversification of NM applications in various fields, such as agriculture, electronics, biomedicine, catalysis, and bioremediation, to name a few, enabled by their unique properties when compared to their bulk or molecular counterparts [2,3,4,5]. The difference between bulk and NMs is related to the interaction and behavior of surface atoms at the nanoscales that are less stable and, therefore, more reactive [6]. Moreover, the proportion of surface atoms increases with average binding energy [6]. Besides, the surface-to-volume ratio increasing inversely to the size confers characteristics controlled by surface properties, similar to free atoms [6]. In the same way, Van der Waals and electromagnetic forces become predominant in NMs surpassing gravity and inertia [1]. All these peculiarities taken together yield NPs’ unique optical, electronic, electrical, mechanical, chemical, thermal, and magnetic properties [1].

Nanomaterials are classified according to their dimensionality, morphology, state, and chemical composition [1,7]. Based on dimensionality and shape, NMs are commonly divided into four categories [1,2]. Zero-dimensional (0-D) NMs, such as nanospheres, nanocubes, polyhedral nanoparticles (NPs), and quantum dots (QDs), have all their dimensions at the nanoscale (1–100 nm) [2]. One-dimensional (1-D) NMs have two dimensions at the nanoscale; these include, for example, nanorods, nanotubes, nanowires, and nanofibers. Two-dimensional (2-D) NMs have only one dimension at the nanoscale, such as nanofilms, nanoplates, and nanocoatings [2]. The last category consists of three-dimensional (3-D) NMs that have their three dimensions beyond 100 nm and result from a combination of multiple NPs [1]. Foams, carbon nanobuds, nanoflowers, polycrystals, and honeycombs are examples of 3-D NMs [2,3,8].

Several methods enable the synthesis of NPs; they are divided into two general categories: bottom-up and top-down methods [8]. Bottom-up approaches rely on atoms and molecules to build up the NMs [8]. This route includes the sol-gel process, hydrothermal route, pyrolysis, and biosynthesis [2]. On the other hand, top-down approaches consist in downsizing bulk materials into corresponding NMs [2] as are mechanical milling, nanolithography, laser ablation, sputtering, and thermal decomposition [2,3,8].

Bottom-up synthesis of NPs has been explored for many decades and is the most preferred route by researchers due to high yields, relatively low energy requirements in comparison with physical approaches, and offers excellent control over the NP size and morphology [9,10]. Usually, chemical routes do not require sophisticated equipment nor highly skilled professionals [11,12,13,14]. Although there are numerous advantages and applications for NPs produced by chemical routes, existing limitations still restrict their integration into marketed products for many reasons: (i) the use of toxic reagents during the processes makes the products highly toxic or not totally meeting the biocompatibility standards for the generation of dangerous byproducts; (ii) high production costs due to expensive reagents employed, and (iii) uncertainties related to the fate of these NMs in the long term [10,15]. Therefore, the design of novel methods is a key challenge in nanotechnology to overcome these limitations and give rise to biocompatible NMs with unique properties. In this sense, the biosynthesis of NMs, also known as bio-nanotechnology, constitutes a serious alternative as it has witnessed tremendous developments over the last decades. This relatively new “green approach,” in comparison with chemical and physical methods, has been shown to be environmentally friendly and cost-effective [3,16,17], and yields NMs that are highly biocompatible exhibiting interesting physicochemical properties [15,18,19,20]. Although NP biosynthesis has been hailed as a promising route for the fabrication of valuable NMs, more research is still needed to understand the underlying mechanisms, control morphology, enhance the colloidal stability, ease the surface functionalization, maximize production yields, and achieve scalability.

Generally, a binary chalcogenide material refers to an inorganic, solid compound made of a transition metal cation, such as cadmium, copper and zinc, and a chalcogen anion, specifically sulfur, selenium, and tellurium, yielding sulfide, selenide, and telluride materials, respectively [21,22]. In addition to ternary and quaternary chalcogenides [23,24], it is also possible to fabricate core-shell chalcogenide NMs [25,26]. QDs constitute a special class of chalcogenides that are less than 10 nm in size. Besides being used in several fields, such as electronics, energy, and solar panels [22,23,25,26,27], QDs have attracted tremendous attention among materials’ scientists for various applications in the biomedical field [28,29,30]. In fact, QDs offer superior characteristics when compared to organic fluorophores. They offer a very high quantum yield and possess a broad absorption with narrow symmetric emission spectra that span from the ultraviolet (UV) to the near infrared region, owing to a size-emission dependence [28]. In other words, tiny QDs tend to emit colors in the blue region. As the QD size increases, its emission redshifts. Besides, QDs are very photostable and resistant to photobleaching and chemical degradation.

Owing to their unique properties and combined with the adequate surface functionalization, QDs have been widely exploited in the biomedical field [28,29,30,31]. For instance, they are used to image live cells [32], efficiently label different cellular components and proteins, either located inside the cell or at its surface [30,33], image deep tissues [34], and synchronously image and treat cancer by acting as a drug carrier that also monitors the delivery process [35]. Besides, semiconductor nanomaterials have been widely investigated for applications in different bioimaging modes and emerging approaches for cancer diagnostics and therapy [36,37,38].

Among synthesized chalcogenides, sulfides hold a prominent place owing to their interesting applications and sulfur availability in nature, in contrast with selenium and tellurium that are very scarce and, hence, more expensive. Several compounds may also serve as the source of both sulfur and metal, such as CdSO_2_ and ZnSO_2_. The present work reviews the multiple achievements regarding the synthesis of sulfur-based chalcogenide nanoparticles (S-NPs) relying on different bio-entities, namely bacteria, fungi, yeast, algae, plants, biomolecules, and viruses. Besides the impact of experimental parameters, the most important aspects and peculiarities of involved mechanisms are discussed to comprehend the processes that rule the synthesis of the S-NPs and control their characteristics which, in turn, confer on them interesting properties and make them attractive candidates for various applications in several disciplines. Finally, crucial findings are summed up and hints for future developments are detailed.

## 2. Biosynthesis of Sulfur-Based Nanoparticles

The biosynthesis of NMs is a bottom-up approach that consists in challenging natural bioresources by different salts to produce different types of NPs [17,39,40] whose properties are of paramount importance since they display high stability, water solubility, and, most importantly, improved biocompatibility, which are key assets for their application in the biomedical field and in the environment [41,42]. Over the last decades, various NMs have been biosynthesized, typically metallic [43,44], metalloid [45], oxides [46,47,48,49], carbonates [50,51], and chalcogenide [52,53]. On the other hand, countless studies have reported the biosynthesis of NMs using various biological entities [54,55], such as bacteria and actinomycetes [10,56,57], fungi and yeast [10,58,59,60,61], plant extracts [62,63], algae [64,65], viruses [66], and biomolecules [67,68,69].

Despite the immense achievements in bio-nanotechnology in terms of produced NMs (chemical nature, composition, morphology), used bio-entity and derivatives (bacteria, fungi, biomolecules, etc.), experimental setup (reagents’ concentration, pH, temperature), essential knowledge is yet to be generated to fully elucidate and understand the governing mechanistic aspects of these bioprocesses, to enable tight control over the NP properties and production.

**Figure 1:**
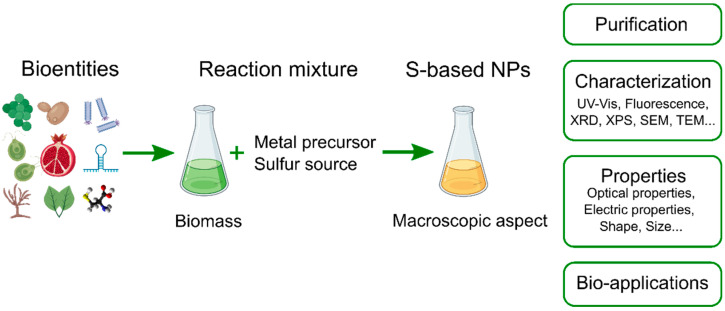
A schematic of the biosynthesis of sulfur-based nanoparticles (S-NPs). First, the biomass is generated starting from a biological resource. Then, this biomass is challenged by the metal precursor and, in some cases, the sulfur source and any other relevant reagents. This gives rise to S-NPs that are purified and characterized. Finally, the bio-applications of these NPs are studied.

Figure 1 illustrates the general processes of the S-NP biosynthesis. Usually, this process begins with biomass generation where organisms are grown in culture media under adequate conditions to produce the necessary biomass [17,70,71,72]. This biomass is then appropriately treated to yield the desired biomass for the research purpose, such as, for example, isolated cells, cell-free extracts, whole living cultures [52,73], plant extracts [74,75], isolated biomolecules [76,77], and viruses [78]. Subsequently, metal precursors and, sometimes, sulfide salts and other relevant reagents are added at the adequate concentrations to, usually, aqueous solutions containing the desired amount of the biomass; then, the experiment is carried out at set parameters (e.g., pH, reaction time, temperature, etc.). In many cases, the synthesis is performed without a sulfur source, as the bio-entity provides it through mechanisms discussed in the following sections [79,80,81]. In most cases, the completion of the NP biosynthesis is accompanied by a visible color change of the reaction medium. Finally, the as-obtained S-NPs are purified for characterization and potential application.

### 2.1. Mechanisms of S-NP Biosynthesis

#### 2.1.1. Intracellular Synthesis

Rai et al. described a general hypothetical mechanism of the intracellular biosynthesis of metallic NPs using fungi that consists of three principal steps: trapping, bio-reduction, and synthesis [82]. During the trapping, electrostatic interactions occur due to the cell surface negative charge and the ion positive charge. Then, the bio-reduction occurs as biological molecules, like enzymes and amino acids, reduce the metal ions to yield, finally, the NP formation [82]. In microbes, dissolved ions enter the cell through the magnesium or manganese transfer system; then, the NP formation occurs in the cytoplasm due the activity of intracellular enzymes and peptides [83,84,85]. However, this mechanism can only apply partially to S-NPs biosynthesis since sulfates may undergo a chemical reduction while the oxidation state of the metal cation remains unaffected. In the case of the intracellular NP generation, extra recovery and purification processes might be required leading to high production costs. First, the NP recovery through cell lysis is commonly carried out; this destroying procedure prevents the use of the cells for another NP production cycle. The recovered NPs are then purified using several techniques, such as centrifugation, freeze-thawing, and ion exchange columns [15].

#### 2.1.2. Extracellular Synthesis

The extracellular synthesis of NP refers to processes occurring outside the cell [10,86]; it uses a cell-free extract or in the presence of the cells [77,87,88,89]. Compared to intracellular approaches, the living cells could theoretically be recycled indefinitely to carry out NP fabrication; besides, the NP recovery is easy, simple, and fast, resulting in very competitive costs and large-scale production opportunities [90,91]. Typically, aqueous solutions of corresponding salts, such as silver nitrate and cadmium chloride, are introduced into cell suspensions where they interact with enzymes located at the cell membrane and/or excreted biomolecules [81,92,93,94] to yield NPs in suspension or absorbed on the cell membrane [90].

The biosynthesis of S-NPs may rely on only metal precursors without a sulfur source or both metal and sulfur precursors [90]. In the first case, bacteria, yeast, and fungi are commonly used. Although the mechanism is still unclear, many authors argue that the presence of metal ions in the medium activates the production of sulfides (S^2−^) by the organisms to detoxify the medium, resulting in the formation of metal-sulfide NPs [83,84,95,96,97]. Sulfide synthesis by these organisms occurs via two pathways: assimilatory and dissimilatory sulfate reduction. Both are anaerobic processes that use sulfate (SO_4_^2−^) as the starting material to obtain energy [90,95].

#### 2.1.3. Dissimilatory Sulfate Reduction

**Figure 2:**
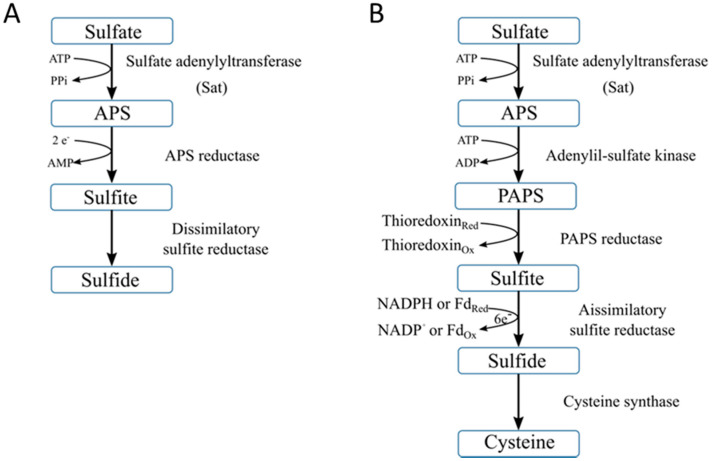
Sulfate reduction pathways in bacteria. (**A**) Dissimilatory sulfate reduction (DSR); and (**B**) assimilatory sulfate reduction (ASR). Adapted from Ref. [95] published by MDPI Cells under an open access Creative Commons (CC) by license.

Dissimilatory sulfate reduction (DSR) is an anaerobic pathway of specialized bacteria, known as sulfate-reducing bacteria (SRB) [95,98]. In this process, sulfate is used as a terminal electron acceptor and energy source [95,98,99]. This pathway can be divided into three main steps [95]. First, the sulfate must be activated before the cell uptake. The enzyme sulfate adenylyltransferase (Sat) reduces the sulfates into adenosine-5-phosphosulfate (APS) and generates pyrophosphate (PPi) as the co-product. In the second step, the enzyme APS reductase reduces, inside the cells, the APS to sulfite (SO_3_^2−^) and adenosine monophosphate (AMP). Finally, the enzyme dissimilatory sulfite reductase reduces the sulfite to sulfide to form hydrogen sulfide as a terminal product that is released by the SRB to the medium [95,98,100]. Figure 2A represents a schematic description of the DSR process.

#### 2.1.4. Assimilatory Sulfate Reduction

The assimilatory sulfate reduction (ASR) pathway is mainly performed by bacteria and plants. This pathway uses sulfate to produce cysteine, an essential amino acid, as the final product (Figure 2B) [95]. Similar to DSR, sulfate is first activated by Sat enzyme and reduced to APS which is then reduced to 3-phosphoadenosine-5-phosphosulfate (PAPS) by adenylyl-sulfate kinase [95]. PAPS is then reduced by PAPS reductase to sulfite, which is subsequently transformed to sulfide and finally cysteine. These two reactions are catalyzed by assimilatory sulfite reductase and cysteine synthase, respectively.

#### 2.1.5. Metal Sulfide Nanoparticle Biosynthesis Using Metal and Sulfide Precursors

DSR and ASR pathways are followed to produce sulfide-based chalcogenide NMs using living organisms when only metal salts are supplied [90,101]. On the other hand, it is suggested that if metal and sulfide salts are added to the culture medium, the toxicity caused by metal ions induces the organisms to produce or excrete proteins and other peptides that bind them and successively interact with sulfide anions. Consequently, less toxic particles are formed [96,101]. The excretion of biomolecules and their binding to metal ions are also associated with size control, stabilization, and properties of the biosynthesized NPs [93,102,103,104].

### 2.2. Biosynthesis of S-NPs Using Microorganisms

Bacteria, fungi, yeast, and microalgae have been extensively used to produce inorganic NPs [16,90,91,101] due to their high tolerance and adaptability to environments with high levels of metal ions [84,105]. Stress conditions activate the detoxification mechanisms, such as ASR and DSR pathways, and produce other proteins, like metallothioneins, phytochelatins, and glutathione-related peptides [83,84,96,106,107]. These peptides chelate metal ions to give rise to metal–peptide complexes. Specifically, metal ions interact with the sulfide groups of peptides to form coated metal–sulfide crystals [105,108,109,110]. Additionally, the above-mentioned microbes should be further explored due to their easy cultivation at ambient temperature, atmospheric pressure, mild pH, and relatively fast growth that may last from a few hours to a few days [15].

#### 2.2.1. Using Bacteria

Bacteria have been extensively used in NM biosynthesis. Among the biosynthesized S-NPs, cadmium sulfide (CdS) NPs are the most reported in the literature, as depicted in Table 1. In 1993, Cunningham and Lundie were the first to report the biosynthesis of spherical CdS NPs utilizing *Clostridium thermoaceticum* and showed the dependency of this process on energy [96]. In two sets of bacterial cultures, cadmium chloride and cysteine were added to the culture media, but glucose, an energy source, was added to only one group. As a result, CdS NP formation occurred exclusively in the non-starved cells after 12 h as evidenced by a color change to bright yellow and transmission electron microscopy (TEM) micrographs. On the other hand, the starved cells did not produce CdS NPs 7 days later. However, NP formation started 24 h after supplying glucose. Additionally, sulfide production was about 4-times higher in cultures challenged by cadmium cations compared to controls. Similarly, the activity of cysteine desulfhydrase started early in the reaction solution containing the metal ions, suggesting that the cadmium presence activated its expression. This resulted in sulfide production via cysteine desulfhydration, thus reducing the medium’s toxicity.

In 1997, Holmes et al. reported the extracellular biosynthesis of CdS NPs of 5–200 nm in diameter using *Klebsiella pneumoniae* [83]. Although the biomass was challenged by different precursors, such as those of lead, zinc, mercury, copper, and silver, NPs made of CdS were the only resulting product. This might be explained by the greater production of sulfide ions that was achieved exclusively in the presence of 2 mM of Cd^2+^. Using the same species, CdS NPs were fabricated and their photocatalytic activity was evaluated [97]. As a result, these biogenic CdS NPs behaved similarly during the photoreduction of methyl viologen (MV^2+^) and methyl orange (MO^−^) when compared to their chemically produced analogs. Additionally, *K. pneumoniae* was used to produce spherical CdS and zinc sulfide (ZnS) NPs whose antimicrobial activity against pathogens, such as *Streptococcus* sp., *Staphylococcus* sp., *Lactobacillus* sp., and *Candida albicans*, was assessed [84]. According to the well diffusion method results, CdS and ZnS NPs considerably inhibited the growth of these pathogens. Recently, Carrasco et al. used the lithobiontic bacterium *Pedobacter* for the extracellular biosynthesis of spherical CdS NPs of 3 nm in size [126]. Exploited as photosensitizers in solar panels, the as-produced QDs showed similar capabilities as other biogenic QDs [103].

Different strains of *Escherichia coli* have been investigated for the biosynthesis of CdS NPs (Table 1). For instance, Sweeney et al. tested four strains; however, only ABLE C and TG1 *E. coli* strains, after being incubated with cadmium chloride and sodium sulfide, gave rise to spherical and elliptical CdS NPs of 2–5 nm in size and crystallizing in the wurtzite structure [117]. Moreover, the nanocrystal formation depended on the bacterial growth phase as CdS NPs were produced about twenty-fold more in the stationary phase compared to the mid-logarithmic phase. Besides, it was hypothesized that glutathione content plays an essential role in the intracellular growth of the NPs [117]. Recently, Tian et al. relied on glucose to increase the synthesis rate of CdS_x_Se_1−x_ QDs of ~2 nm in size using *E. coli* JM109 [132]. This effect was attributed to an enhanced synthesis of NADPH and reduced thiol (-SH) groups that directly improved the tolerance to metal toxicity and NP mineralization. It is also important to note that different strains of *E. coli* may promote the biosynthesis of CdS NPs via either intracellular [117] or extracellular pathways [122].

The genetic engineering of bacteria has been investigated for decades for multiple applications, including insulin production, cancer treatment, and bio-nanotechnology [133,134,135]. By inserting specific gene sequences, mutant bacteria overexpress certain biomolecules or favor given metabolic pathways involved in the biosynthesis of S-NPs [135,136]. For instance, the mutant *E. coli* strain JM109 that increases its phytochelatin (PC) production by 10-fold synthesized CdS NPs of 2–6 nm in size due to the different PC populations that act as the template [124]. In the same study, after being genetically modified to produce PC4, a particular PC type, *E. coli* strain R189 promoted the synthesis of uniform, 3–4 nm diameter CdS NPs [124]. Besides, higher NP yields were achieved employing the recombinant *E. coli* ABLE C that overproduces glutathione synthetase (GS), an enzyme involved in the generation of glutathione, a key molecule in the synthesis of CdS NPs in yeast [120]. In this case, the yield was 2.5 times higher compared to the wild-type strain although the size and crystallinity remained unaffected. In another approach, *E. coli* BL21 was genetically engineered to produce CDS 7, a peptide capable of binding CdS precursors to form QDs. As a result, the obtained CdS QDs exhibited an enhanced stability and a better control on the particle size averaging 6 nm, when compared to their counterparts produced by the wild-type strain [121].

Sulfate-reducing bacteria (SRB) are anaerobic microorganisms able to degrade organic compounds using sulfate as the terminal electron acceptor [95,98]. They are widely studied due to their impact on the natural sulfur cycle and waste water treatments [98]. Besides, SRB have attracted significant attention for the biosynthesis of S-NPs owing to their high capacity to produce sulfide ions via the DSR pathway [90,101]. For example, Qi et al. reported the intracellular and extracellular production of CdS NPs using *Desulfovibrio caledoiensis* that was highly related to SRB metabolic sulfide production since a linear relationship was established between the fluorescence intensity of the bio-produced NPs and bacteria concentration [114]. In fact, high SRB concentrations produced elevated sulfide amounts through their metabolism [114]. Furthermore, these CdS NPs were successfully employed to detect SRB [114]. Similarly, using *D. alaskensis* yielded the intracellular and extracellular biosynthesis of CdS NPs of 10–46 nm in size that were either cubic or hexagonal depending on the precursor concentration [115]. In a recent study, the effective removal of EDTA-chelated cadmium ions from the medium of *D. desulfuricans* resulted in the extracellular precipitation of spherical CdS NPs of 40–80 nm in diameter. These NPs exhibited a high photocatalytic activity in the degradation of rhodamine B [116].

In addition to CdS NPs, other S-NPs, such as those of zinc sulfide (ZnS) and lead sulfide (PbS), have also been synthesized using SRB [100,137,138,139]. For instance, Labrenz et al. reported sphalerite ZnS NP formation within biofilms of *Desulfobacteriaceae* sp. [138]. The observed nano-aggregates were 2–5 nm spheres. Another study reported the precipitation of sphalerite and wurtzite ZnS nanocrystals with sizes of 12–14 nm and ~48 nm, respectively [139]. This is an in-situ study made in peatlands suggesting that the primary organisms participating in this process are SRB as dissimilatory sulfite reductase (dsrAB) genes were found using polymerase chain reaction tests. Moreover, controlled experimental methodologies have been implemented for the bio-production of NPs using the SRB *Desulfotomaculum* sp. that act as biofactories for the controlled crystal growth of PbS NPs at different pH ranges and temperature conditions (vide infra) [137]. Additionally, the SRB *Clostridiaceae* sp. promoted the extracellular biosynthesis of PbS NPs with cuboidal, nanosheet, and spherical morphologies; this process was primarily mediated by optimizing polyethylene glycol (PEG) concentration in the cell cultures demonstrating, thus, favorable conditions for the easy and low-cost NP purification [100]. In another study, Murray et al. reported a cell-free approach for ZnS NP production using *D. desulfuricans* in which the off-gas hydrogen sulfide (H_2_S) from SRB was injected into Zn^2+^ solutions to produce whitish solutions indicating ZnS NP formation that was confirmed by XRD analysis [87]. The bio-produced ZnS QDs were spherical with a diameter of ~2.4 nm and crystallized in the cubic system. On the other hand, da Costa et al. fabricated 20–30 nm ZnS NPs by mixing Zn^2+^ and *D. desulfuricans* sulfide-rich supernatant, in the presence of TiO_2_ and SiO_2_ powders that served as ZnS precipitating substrates [101]. Qi et al. described a controlled biosynthesis of ZnS QDs employing *Clostridiaceae* sp. Their nucleation, growth, and crystalline system, i.e., sphalerite vs. wurtzite, were highly influenced by the amount of hydroxypropyl starch (HPS) added to the culture medium [140]. The authors think that HPS serves as a matrix for NP formation, and, combined with EDTA, contributes to the easy NP purification.

Qi et al. achieved a high-yield, pure extracellular and continuous biosynthesis of ZnS QDs that could reach a monthly production of 35−45 g L^−1^, extrapolated from the one-week results [104]. They used a mix of the SRB *Desulfovibrio* sp., *Clostridiaceae* sp., *Proteiniphilum* sp., *Geotoga* sp., and *Sphaerochaeta* sp., to produce highly photoluminescent ZnS QDs of 6.5 nm in size. Besides, Fourier-transform infrared spectroscopy (FTIR) evidenced that this formation was greatly influenced by SRB secreted proteins which, in addition to elevated sulfide synthesis, were the main contributing factors to the obtained QDs’ size and shape. Furthermore, the same team reported the bioproduction of CdS, PbS, and copper sulfide (CuS) QDs. ZnS NP biosynthesis has also been achieved using *Serratia nematodiphila* [141], mixed SRB [142], and other bacteria belonging to *Thermoanaerobacter*, *Rhodobacter*, *Pseudomonas*, *Bacillus*, *Enterococcus*, and *Lactobacillus* genera (Table 2). Lastly, Pb_3_O_4_ material, made by several strains of bacteria, is responsible for the red stains that appear on marble; however, the characteristics of this oxide were not made available [143].

Bacteria isolated from extreme environments, such as halophilic bacteria, have also been investigated for their ability to produce S-NPs [70,111,130]. For instance, Antarctic *Pseudomonas* spp., a species resistant to both peroxide and cadmium chloride, produced CdS NPs of 10–40 nm in size at 15 °C in a time-dependent fluorescence response, changing from green to red as the size increases [130]. Knowing that sulfide production is essential for the biosynthesis of CdS QDs in the presence of cysteine owing to the action of cysteine desulfhydrase enzyme, CdS NP production was detected as early as two hours after starting the reaction using three strains while the last one, *P. fragi* ATTC 4973, could not produce H_2_S in the set conditions and, hence, formed no NPs [130]. In another study relying on *Acidithiobacillus* spp., the addition of inorganic phosphate (Pi) to the growth medium enhanced the intracellular biosynthesis of CdS QDs at pH 3.5 as Pi may help the cells overcome the toxicity and be involved in Cd^2+^ co-transportation [111]. This finding was corroborated by other findings [52,111,119].

It is also possible to obtain CdS NPs using solely the metal precursor as described using Antarctic species, namely *Pseudomonas*, *Psychrobacter*, and *Shewanella*, cultured in a sulfur-free medium [129]. For instance, Gallardo et al. detailed the CdS QD biosynthesis employing the Antarctic species *P. fragi* GC0 and assessed the impact of the sulfur source, specifically sulfate, sulfite, thiosulfate, sulfide, cysteine, and methionine, on the synthesis pathway. As a result, CdS QDs formed inside the cells in the presence of all sulfur salts while the extracellular biosynthesis occurred in the presence of cysteine and methionine [80]. The medium osmolarity also impacts the bacterial ability to produce CdS NPs. For instance, the salt-resistant halophilic species *Halobacillus* sp. DS2, isolated from Atacama Salt Flat, Uyuni Salt Flat, and the Dead Sea, promoted the biosynthesis of stable CdS QDs at different NaCl concentrations (0–8%) while the control species, *E. coli*, could not since it was unable to produce H_2_S at NaCl concentrations higher than 4% [41].

Recently, core/shell NPs were synthesized for the first time using bacteria. Specifically, CdS/CdSe core/shell NPs were bio-produced using *E. coli* and exhibited better photovoltaic response when compared to biogenic CdS QDs obtained using the same species [122]. Thus, the biosynthesis route could be an efficient alternative to chemical methods for the generation of novel photovoltaic materials. In addition to CdS and silver sulfide (Ag_2_S) QDs, ternary CdSAg QDs were produced for the first time using *E. coli* [103]. First, CdS QDs were produced in the presence of Cd^2+^ and cysteine for different reaction times, resulting in fluorescent NPs of different colors: green, yellow, and red as the size increased with longer reaction times (10, 40, and 110 min, respectively). Then, ternary CdSAg QDs were synthesized through cation exchange, starting with previously obtained CdS QDs and AgNO_3_ at different concentrations, an occurrence exclusive to *E. coli*. Fluorescence spectra indicated the existence of an isosbestic point (IP) at 25–40 μM Ag^+^ concentration, indicating the formation of CdSAg QDs. These findings expand the possibilities offered by microbes for the production of high added-value nanomaterials and constitute an incentive for further exploration.

There are two particular forms of biogenic iron sulfide NPs: greigite (Fe_3_S_4_) and pyrite (FeS_2_), mainly produced by magnetotactic bacteria (MTB) via a natural biomineralization process that is part of their metabolism [160,161,162,163]. In 1990, Mann et al. reported biogenic rhombohedral and hexagonal greigite and pyrite nanocrystals of ~75 nm in size inside an MTB collected from sulfide-rich brackish sites [161]. Similarly, greigite NPs with a size distribution of 50–90 nm were found inside MTB [162]. Besides, SRB [163] and *Actinobacter* sp. [160] promote the biosynthesis of iron sulfide (FeS), greigite (Fe_3_S_4_), and pyrite (FeS_2_) NPs. On the other hand, the extracellular biogenesis of PbS nanocrystals has been implemented using *Rhodobacter sphaeroides* [79] and *Shinella zoogloeoides* [150]. These studies demonstrated the involvement of cysteine desulfhydrase in H_2_S production and the subsequent NP formation. Cystathionine γ-lyase (smCSE) is another enzyme linked to the crystallization of PbS, CdS, and PbS/CdS core/shell NPs using *Stenotrophomonas maltophilia* [76,77]. This enzyme degrades L-cysteine into ammonia (NH_3_), pyruvate, and H_2_S, the latter serving as the sulfide source during the S-NP synthesis.

Bacteria have enabled the biosynthesis of other types of S-based chalcogenide nanocrystals. For instance, bismuth sulfide (Bi_2_S_3_) NPs were produced using *Clostridiaceae* sp. following a “water–oil two-phase” procedure [157]. This yielded nanorods and nanoneedles depending on lactic acid and sulfate (SO_4_^2−^) concentrations (vide infra). In another approach, hexagonal Bi_2_S_3_ NPs were extracellularly synthesized using *Clostridium acetobutylicum* [158]. Their dimensions were time-dependent as cultures incubated for 5 days produced NPs of 6–10 nm while cultures incubated for 7 days produced crystals of ~440–500 nm. Lastly, the biogenesis of different S-based chalcogenide NPs, such as spherical silver sulfide (Ag_2_S) NPs [152,153], arsenic-sulfide nanotubes [155,156], and copper sulfide (CuS) nanorods [159], has also been described (Table 2).

#### 2.2.2. Using Yeast and Fungi

Fungi and yeast have proven effective in bio-nanotechnology via various, low-cost and scalable approaches that result in the production of diverse nanomaterials [16,45]. In comparison to bacteria, fungi possess greater tolerance to metal ions due to higher cell wall binding capabilities enabling more metal bioaccumulation and, therefore, effective and large-scale NP bio-production [10,20,92,101]. In fungi, including yeast and plants, metal ions trigger different mechanisms for the enhanced synthesis of metallothioneins (MTs) or glutathione-derived peptides, also known as phytochelatins, that chelate these cations, thus decreasing the medium toxicity [107,164]. MTs are translated from mRNA while PCs are enzymatically derived from glutathione (GSH, γ-Glu-Cys-Gly) and have (γ-Glu-Cys)_n_-Gly as their general structure where n = 2–11 [106,107]. The mycosynthesis of different sulfur-based chalcogenide NPs has been achieved using several species, such as *Fusarium oxysporum* [165,166], *Coriolus versicolor* [167], *Saccharomyces cerevisiae* [125,168], and *Trichoderma harzianum* [169], as summarized in Table 3. For clarity, yeast is treated separately although it is part of the fungi kingdom.

##### Yeast

The first demonstration of NP biosynthesis using fungi, more specifically yeast, was presented in 1989 by Dameron et al. who synthesized monodisperse quantum CdS crystallites using the yeasts *Candida glabrata* and *Schizosaccharomyces pombe* [108]. The authors found that short peptides, determined to be PCs, controlled the intracellular biosynthesis of the NPs and stabilized them by acting as a coat [108,110]. Similar results were obtained using *C. glabrata* [105,109]. Although the fluorescent CdS NPs formed intracellularly, they were coated either by PCs or other glutathione-related peptides, depending on the nutrients contained in the culture medium.

In fed-batch conditions, Krumov et al. carried out the synthesis of CdS NPs mediated by two species, *S. pombe* and *C. glabrata*. The process was intracellular for both and depended on glucose uptake as the energy source, but *S. pombe* exhibited a higher yield [170]. In another study, CdS QDs of 2–6 nm in size were produced using the same species [175]. *S. cerevisiae* also generated spherical CdS NPs of 2.2–4.5 nm in diameter [125] or smaller (~2 nm) [168]. Lastly, PbS nanocrystals were made using *Rhodosporidium diobovatum* [179], and *Torulopsis* sp. [176], as described in Table 3.

Overall, S-NP biosynthesis using yeast is intracellular in most cases as a response to the stress induced by the presence of metal ions, like cadmium, zinc, and lead, that consequently triggers the detoxification mechanisms, resulting in the sequestration of these cations in a less-toxic form, i.e., as S-NPs, inside the cell [105,170]. Moreover, all studies reported the NPs to be spherical. Besides, yeast-mediated S-NPs are almost in all instances less than 5 nm in diameter and might be considered QDs. This peculiarity is ascribed to the peptide shell that surrounds the NPs, preventing their aggregation and hindering their morphology from evolving, therefore, resulting in tiny, colloidally-stable S-NPs [170,179].

##### Fungi

Ahmed et al. proved that *F. oxysporum*, immersed in a CdSO_4_ solution, secretes sulfate reductase enzymes to produce sulfides and convert metal ions into CdS NPs [165]. Moreover, Uddandarao and Mohan showed that ZnS QDs, obtained extracellularly using *Aspergillus flavus*, are capped and stabilized by protein residues rich in cysteine and methionine [71]. The authors suggested that metal ions stressed the cells, thus activating the ASR pathway and ZnS NP formation. These NPs exhibited an interesting antimicrobial activity against *E. coli* since their interaction with the cell wall induced the generation of reactive oxygen species (ROS) that caused irreversible cell damage. The biogenesis of CdS NPs without any added sulfur precursor was demonstrated using *C. versicolor* immobilized in a continuous column. As the cadmium solution passed through the column, a yellow color appeared indicating the formation of spherical CdS NPs of 8–15 nm in size [167]. The biosynthesized NPs described in these studies were highly stable owing to the presence of proteins and peptides attached to their surface [71,169,172,177,179].

Besides CdS NPs, *F. oxysporum* can also produce spherical CuS NPs of 2–5 nm in diameter [183]. However, their hydrodynamic diameter reaches 20 nm due to the formation of an entrapping peptide shell. Following a novel approach, CuS NPs were fabricated using the same species; however, the application of electric currents of less than 20 mA to prevent any lethal consequences to the organisms improved the yield by ~three-fold in comparison to control samples [81]. Although the applied electric currents hindered their growth, the cells consumed higher amounts of glucose owing to DNA changes, produced more proteins, and increased their cell wall permeability. Furthermore, Ag_2_S NPs synthesized using *Pleurotus ostreatus* presented good antimicrobial effects and luminescent properties in the green region [182].

Unlike yeast, fungi promote the biosynthesis of S-NPs exclusively via an extracellular process as this happens with other types of NPs [184]. Like yeast, all nanocrystals are spherical in shape although their size usually exceeds 5 nm. From a composition point-of-view, fungus-mediated S-NPs exhibit a large variety of compounds as CdS, ZnS, PbS, Ag_2_S, and CuS NPs are formed. Interestingly, the same NM might be produced using several species. For instance, CdS NPs are obtained using *A. niger* [92], *F. oxysporum* [165,171], *Trametes versicolor* [177], and *T. harzianum* [169]. On the other hand, the same species may promote the formation of different S-NPs. For instance, *F. oxysporum* produces CdS [165], ZnS [166], and CuS NPs [81].

Considering their ability to overcome metals’ toxicity by releasing different peptides and enzymes, high tolerance to metal ions and bioaccumulation capacity, fungi and yeast are attractive candidates for large-scale production of S-NPs, especially since they yield more NPs than other entities, such as bacteria and plants [101,184]. Although genetic engineering tools aiming at overexpressing specific components involved in the synthesis of S-NPs constitutes a tremendous asset, post-translational modifications remain challenging when compared to simpler organisms, like bacteria [185]. Several factors make the design of permanent bioreactors based on yeast and fungi for the production of S-NPs challenging. In fact, cell lysis is needed to recover the NPs when yeast is used as the synthesis is most often intracellular. On the other hand, the presence of precursors is necessary when employing fungi to produce released enzymes and peptides that participate in the extracellular NP synthesis. An additional complication lies in the variability of the NP synthesis kinetics as the process may complete within a few hours or last up to several days, depending on several parameters, such as the used organism and species, its growth stage, and sufficient production of key biomolecules involved in the process [92,168,169,175,178,180,182].

#### 2.2.3. Using Algae

Algae are unicellular or multicellular organisms belonging to four domains/kingdoms: Bacteria, Plantae, Chromista, and Protozoa, and are commonly found in moist freshwater surfaces, and aquatic habitats. Although NP biogenesis using algae is a relatively new approach, it is still promising. Algae can produce stable metallic NPs under very mild conditions using whole living cultures or their extracts, e.g., disrupted cells, cell-free supernatant, and isolated biomolecules, to reduce metal cations to their metallic state and the subsequent formation of metallic NPs through bioremediation processes [64,86,186,187]. The first report of NP formation using algae described the production of Au NPs of different shapes using a protein isolated from *Chlorella vulgaris* [94]. Since then, several groups have diversified this approach in terms of, for instance, obtained products, used species, impact of experimental parameters, and elucidation of mechanistic aspects [16,86,94,188,189,190]. Although it is accepted that algae-assisted synthesis of NPs relies on the cell wall biomolecules, peptides, excreted polysaccharides, enzymes, proteins, organelles, etc., further work is still needed to determine the impact of each and establish specific mechanisms to draw the whole picture of these processes [16].

The biosynthesis of S-NPs using algae remains very scarce. To date, only a few examples have been reported [191]. For instance, the intracellular formation of CdS NPs was achieved using the green microalga *Scenedesmus*-24 [192]. The presence of cadmium ions in the medium induced their cell absorption/sequestration. Simultaneously, low molecular weight proteins, such as metallothioneins and phytochelatins, were synthesized. The thiol groups of these proteins interact with Cd ions to form metal-peptide complexes and lower the toxicity. As a result of this detoxification process, CdS crystals of 150–175 nm in size are produced. The blue-green microalga *Spirulina* (*Arthrospira*) *platensis* synthesizes spherical photoluminescent CdS NPs in the sole presence of cadmium nitrate–no sulfur source was added–via an extracellular process [193]. This might stem from Cd^2+^ binding the C-phycocyanin (CPC) biliprotein, the sulfur source provided by *Spirulina*, reported also to bind Hg^2+^ [194]. In fact, a decrease in fluorescence intensity of CPC was observed while cadmium nitrate concentration increased [193]. In this experiment, the algal cells were necessary to enable the biosynthesis of NPs since the presence of Cd^2+^ triggered the release of CPC that, due to their ring-like shape, served as a matrix for the formation of spherical CdS NP of 8–12 nm in size. Besides, Edwards et al. showed that supplementing the green *Chlamydomonas reinhardtii* and the red *Cyanidioschyzon merolae* microalgae with extra sulfate increases their Cd^2+^ tolerance and, concomitantly, CdS crystal production [195]. On the other hand, the cyanobacterium *Synechococcus leopoliensis* appears to be less selective as sulfates, sulfites and cysteine had the same effect. However, the authors did not discuss the features of the obtained CdS crystals. In another study, spherical CdS NPs of ~5 nm in diameter were produced using the cell-free extract of the green microalga *C. reinhardtii* with cadmium chloride and sodium sulfide as cadmium and sulfur sources, respectively [89]. These NPs exhibited interesting catalytic activity in degrading the pollutant dye methylene blue (MB). These few studies show the potential of algal resources in the synthesis of S-based chalcogenide NPs and could warrant further investigations.

### 2.3. Biosynthesis of S-NPs Using Plants

Plants are widely used in bio-nanotechnology. This well-established route relies on cheap, widespread and renewable raw material [101]. Although living plants may promote the in-vivo biosynthesis of nanomaterials [16], the use of plant extracts, such as those of leaves, stems, and fruits, remains, by far, the most explored methodology [63,196]. Unlike their analogs obtained via bacteria- or fungi-mediated processes, NPs produced using plants are sparsely covered by macromolecules; this particularity prevents the molecules from acting as capping agents and substantially altering the NP properties [75]. Several studies have shed light on the NP formation mechanism within live plants [106,164,196]. As the presence of metal cations triggers the generation of reactive oxygen species (ROS) associated with enzyme dysfunction, the plants react by producing stress-related enzymes, such as superoxide dismutase, catalase and peroxidase, to overcome these conditions [106]. In plant tissues, metal cations are chelated by low molecular weight proteins, namely metallothioneins-like proteins belonging to phytochelatins, owing to their thiol groups to yield the NP formation via a similar process to NP synthesis using fungi [106,164]. Using plant extracts, various bio-compounds, such as phenols, terpenoids, ketones, carboxylic acids, aldehydes, enzymes, amides, and flavonoids, are linked to NP synthesis [196].

A few articles describe the plant-assisted biosynthesis of sulfur-based chalcogenide NPs. For instance, Borovaya et al. detailed the formation of spherical CdS QDs of 5.5–6.9 nm in size by challenging extracts of *Linaria maroccana* with CdSO_2_ and Na_2_S for 10 days at 28 °C [197]. Following a similar process, the same group demonstrated the biosynthesis of water-soluble, luminescent spherical CdS QDs of 3–7 nm using the cell suspension of *Nicotiana tabacum* [75]. Their biocompatibility was assessed by challenging *N. tabacum* protoplasts at different concentrations of CdS QDs. As a result, a concentration of 0.012 mg mL^−1^ or lower exhibited no adverse effects to the protoplasts. Following a different procedure, Kaviya et al. obtained spherical CdS NPs using pomegranate peel extract which is rich in phytochemicals that act as capping and stabilizing agents [74]. The CdS NPs produced were excellent detection platforms of Pb^2+^. Moreover, CdS NPs produced using tea (*Camellia sinensis*) leaf extract show antibacterial and anticancer activity in addition to efficient bio-imaging of A549 cancer cells [19]. Taking advantage of their ability to accumulate cadmium and lead metals, the hairy roots of tomato *Solanum lycopersicum* were used to synthesize highly photostable CdS NPs of 4–10 nm [106,175]. This was attributed to Cd^2+^ binding PCs, establishing metal-PC complexes that subsequently resulted in CdS NP formation [175,198]. In another study, the impact of the cellular host choice on biogenic CdS QDs was investigated. As a result, the NPs formed by *S. pombe* were smaller, faster to form in higher yields as Cd^2+^ accumulated in higher amounts in the yeast [175]. On the other hand, the QDs made by *S. lycopersicum* were larger but more photostable.

### 2.4. Biosynthesis of S-NPs Using Biomolecules

The synthesis of NPs using specific biomolecules, such as peptides, proteins, and nucleic acids, is of paramount importance for several reasons: (i) the reagents are known, therefore the identification of the products, especially the NP capping agent, should be easy; (ii) a better understanding of the underlying mechanisms; (iii) importantly, if NPs with interesting properties are obtained, the process reproducibility and scalability would be within reach; (iv) finally, introducing chemical groups of interest to these biomolecules, made easy owing to genetic engineering and synthetic biology, might enhance the process. A few articles have depicted the fabrication of S-NPs via this route. For example, wild-type and mutated transfer RNA, hereafter WT-tRNA and MT-tRNA, respectively, produced spherical CdS NPs of 4–7 nm in diameter after being challenged by cadmium and sulfide ions [199]. In both cases, tRNA acted as a scaffold and ligand. Due to differences in their secondary and tertiary structures, WT-tRNA produced smaller, monodisperse QDs whereas MT-tRNA synthesized larger, polydisperse NPs.

Specific enzymes are known to participate in the production of various NPs [76,77,200]. Sometimes, these are overproduced by the organisms when metal ions are present in the growth medium [85,130]. For instance, cystathionine γ-lyase, an enzyme involved in CdS NP formation [76,77], was isolated and purified from *S. maltophilia* SMCD1, and its ability to crystallize CdS NPs was studied in the presence of cadmium acetate and L-cysteine or Na_2_S [200]. As a result, smCSE catalyzed L-cysteine metabolism to produce sulfide ions and promote CdS formation. Additionally, the impact of sulfide source and capping agent availability on the NP growth kinetics, size, and stability was elucidated. Similarly, the parent enzyme cystathionine, γ-lyase (CSE), isolated from recombinant *E. coli*, and synthesized biocompatible CuInS_2_, (CuInZn)S_2_ and CuInS_2_/ZnS core/shell QDs [201]. CuInS_2_/ZnS core/shell QDs were further conjugated to IgG antibodies and successfully applied in the bioimaging of THP-1 cells that remained alive, thus highlighting the biocompatibility of these nanocrystals. More recently, the enzyme threonine dehydratase, expressed by *P. stutzeri* 273 (psTD), was used to drive the synthesis of CdS nanocrystals of ~84 nm in the presence of L-cysteine which served as the sulfur source and participated in NP stabilization [202]. Additionally, the same enzyme was shown to be essential in bacterial Cd^2+^ resistance since the deletion of its encoding gene resulted in the decrease of H_2_S generation and metal resistance as well as in poorer CdS mineralization. Furthermore, the C-phycoerythrin pigment, extracted from the cyanobacterium *Phormidium tenue*, enables the synthesis of CdS NPs of ~5 nm in size when challenged by CdCl_2_ and Na_2_S [203]. Lastly, rhamnolipids, isolated from *P. aeruginosa*, are used to produce spherical ZnS NPs of 10–15 nm in size [18].

### 2.5. Biosynthesis of S-NPs Using Viruses

Unlike other biological entities, the use of viruses for NP biosynthesis remains limited since additional biosafety and biohazard requirements need to be met, like special isolation techniques, highly skilled professionals and containment infrastructures. These factors elevate the economic costs and, therefore, negatively impact the large-scale production of NP [101]. A handful of methodologies have been designed for NP synthesis using viruses: (i) the virus provides the adsorption surface of metal cations, and their subsequent precipitation resulting in NP growth and NP-decorated capsids [204,205,206,207]; (ii) previously synthesized NPs are mixed with pre-treated viral capsids and allowed to assemble to yield 1-D nanostructures [208]; (iii) the biomolecule-rich viral lysate is challenged by metal cations to elicit their precipitation and give rise to the corresponding NPs [66].

In 1999, Shenton et al. described the formation of CdS nanotubes by nucleating CdS NPs on the surface of tobacco mosaic virus (TMV) in the presence of CdCl_2_ and H_2_S [209]. Owing most likely to the action of the amino acid residues of the capsid proteins, such as glutamate, aspartate, arginine, and lysine, these 5-nm CdS NPs aggregated and accumulated at the TMV surface to form hollow CdS-virion nanotubes of ~50 nm in width, ~16 nm thick electron-dense outer crust and 18 nm diameter internal core. Similarly, PbS nanotubes of 40 nm in width were synthesized using the same virus [209]. In this case, the NPs, either prismatic or irregular, were up to 30 nm in size. Since then, other articles detailed the formation of S-NPs and their precipitation at the surface of either wild-type or genetically engineered viruses to create self-assembled nanostructures envisaged in several applications. For instance, the coat protein pIII of the M13 bacteriophage was found to have an affinity to ZnS in solution. The end pIII protein tethers ZnS and agglomerates with other bacteriophages into a micelle-like structure, thus, ZnS NPs are synthesized inside this arrangement with a particle size of about 2.66 nm [210]. M13 bacteriophage viruses were genetically modified to express the protein pVIII in the capsid, which was found to aggregate CdS and ZnS from solutions, resulting in the formation of nanowires of these two semiconductors [204]. The same year, nanowires based on ZnS and CdS were obtained by expressing the two proteins pIII and pVIII in the same viral capsid [78], like the previous studies [204,209].

## 3. Control over S-NP Biosynthesis

Sulfur-based chalcogenide NPs have been biosynthesized using many organisms and bio-entities as described previously. In fact, using the same bio-organism can sometimes lead to very different NP features, highlighting that many factors, aside from the source, influence the outcomes of the NP production [17]. Slight changes in these variables, such as the temperature, pH, reagents’ concentration, and reaction time, can tremendously affect NP characteristics and properties. Achieving tight control over the NP production process via green approaches in terms of composition, size, shape, colloidal stability, yield, cost, scalability, etc., is of paramount importance since biogenic NPs have exhibited very interesting properties that open them up new avenues in countless applications since they can compete with their counterparts obtained via conventional routes or even surpass them.

NP properties highly depend on factors, such as composition, morphology, stability, purity, and capping agent. Commonly, green-synthesized NPs are formed at ambient to mild temperatures and atmospheric pressures. Importantly, the parameters particularly relevant to consider are the pH, organism, salt sources, biomass concentration, time, and additional reagents [41,100,137,150].

Spherical NPs seem to be easily generated since this morphology is predominant. Many biomolecules concomitantly mediate the synthesis of the NPs and ensure their stabilization by acting as capping agents [16,77,80,95,96,130,186,200]. This results in NPs adopting spherical or round-shaped morphologies. However, no TEM or scanning electron microscopy images are shown in many instances while others display NPs of irregular shapes and large size distribution [88,100,113].

Using the halophilic bacterium *Halobacillus* sp. DS2, Bruna et al. accomplished an interesting control over the biosynthesis of fluorescent hexagonal CdS QDs of 3.6 ± 0.8 nm in size [41]. To that end, the species was grown for 28 days then the cells were harvested and suspended in a buffered aqueous solution at pH 8.8 before being challenged by cysteine and CdCl_2_. Moreover, Chen et al. presented a size-controlled biosynthesis of CdS QDs of 1.5–2.0 nm in size using the white-rot fungus *Phanerochaete chrysosporium*, thioacetamide and Cd(NO_3_)_2_ as S and Cd precursors, respectively, at 37 °C and pH 9–11 for 12 h [172]. Additionally, Gong et al. evaluated the impact of pH and temperature on the synthesis of PbS NPs employing the SRB *Desulfotomaculum* sp. [137]. The optimized conditions to produce high-purity, monophase PbS NPs were found to be pH 6–8 at 30 °C for 48 h of incubation. When the pH increased, the shape evolved from rod to spheroidal. On the other hand, temperature variation in the range of 15–35 °C for a pH fixed at 7 did not affect the NP features. The pH-dependent NP synthesis capability is attributed to the decreased SRB sulfide/sulfate production in alkaline media. The biosorption of metal ions by a given organism is also related to the pH as the cell membrane conformation might be altered, thus, increasing or decreasing its biosorption rate and capacity [150]. Lower pH could protonate the cell surface while alkaline pH might cause the precipitation of metal ions by combining with hydroxyl (−OH) groups [150]. Therefore, pH adjustment can favor or impede the NP biosynthesis since it impacts both the bio-entity and reaction solution [150].

**Figure 3:**
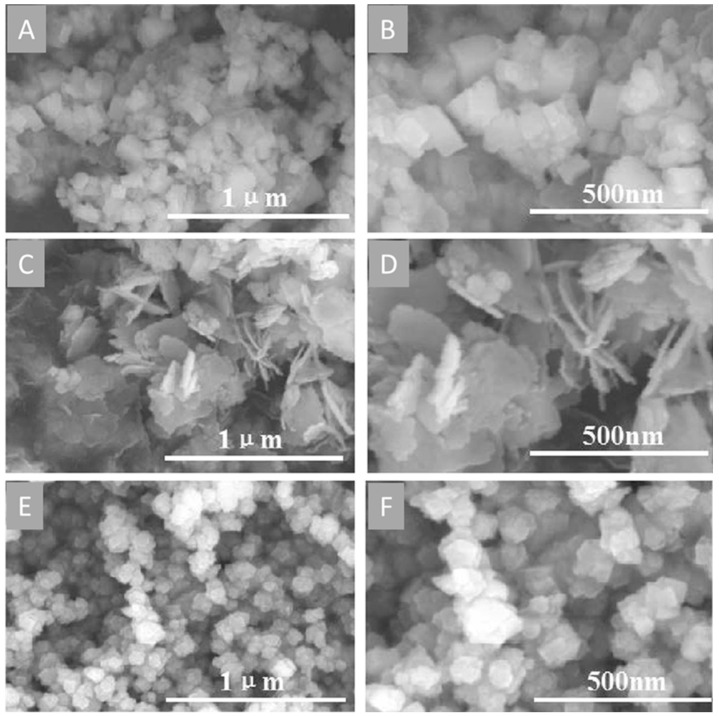
Impact of added polyethylene glycol (PEG) amount on the extracellular biosynthesis of PbS NPs using SRB *Clostridiaceae* sp. studied using scanning electron microscopy (SEM): (**A**,**B**) Cuboidal NPs (4 mM); (**C**,**D**) nanosheets (12 mM); and (**E**,**F**) nanospheres (20 mM). Adapted from Ref. [100] with permission from Springer.

In some cases, the sulfur source or sulfide production by the organisms is the factor altering the size and shape of biogenic S-NPs [100,157,172]. This was illustrated by Yue et al. who used the SRB *Clostridiaceae* sp. to carry out the extracellular synthesis of PbS NPs in the presence of polyethylene glycol at different concentrations: 4, 12, and 20 mM [100]. This resulted in the formation of cuboids (50 × 50 × 100 nm), nanosheets of 10 nm in thickness, and nanospheres of 60 nm in diameter, respectively, as displayed in Figure 3. The authors attributed this effect to the production of sulfide ions (S^2−^) by the bacteria as greater amounts of PEG partially inhibited the cell growth, directly upsetting the sulfur source production and, therefore, the NP morphology. Nevertheless, the interaction nature and strength between PEG and the NP surface should be further investigated to draw a more complete picture of the process.

**Figure 4:**
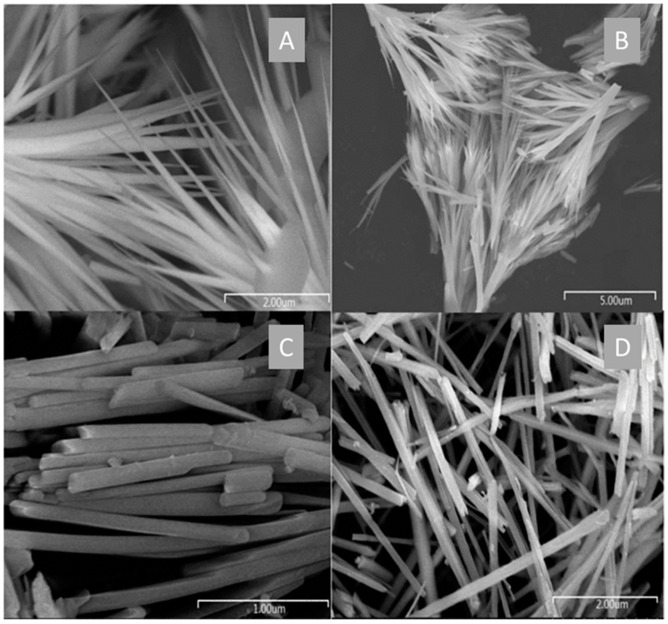
SEM images of Bi_2_S_3_ NPs synthesized by *Clostridiaceae* sp. in the presence of different lactic acid and SO_4_^2−^ concentrations for 20-day incubation: (**A**,**B**) Nanoneedles formed at low dose (0.1 M); and (**C**,**D**) nanorods formed at a high dose (0.3 M). Reproduced from Ref. [157] with permission from Wiley.

Yue et al. demonstrated that lactic acid and Na_2_SO_4_ concentrations directly impact Bi_2_S_3_ NP biosynthesis [157]. As depicted in Figure 4, a large dose of these two reagents (0.3 M) leads to the formation of nanorods whereas nanoneedles are formed at a low dose (0.1 M). The process was performed using the water-oil two-phase system and the synthesis occurred in the water phase. Most likely, the morphology difference was due to different rates of S^2−^ generation promoted by the concentration of both lactic acid and SO_4_^2−^ (from Na_2_SO_4_).

In another study, the crystalline phase of the obtained ZnS NPs varied depending on hydroxypropyl starch concentration. At 0.4 g L^−1^ concentration or lower, the wurtzite phase was favored as the NPs crystallized within the hexagonal system [140]. In contrast, high HPS concentrations (0.8 g L^−1^ or higher) promoted the sphalerite phase as the produced NPs crystallized within the cubic lattice. TEM analysis indicates that the formation of ZnS QDs occurs inside cavities of columnar structures held by HPS. On the other hand, the size of ZnS NPs was inversely influenced by HPS. Typically, QDs of 5.95 ± 1.13 nm in size were produced at HPS concentration of 0.2 g L^−1^ whereas 1.6 g L^−1^ of HPS resulted in QDs of 3.34 ± 0.65 nm in size.

One of the greatest advantages conferred by the green production to NPs lies in their outstanding colloidal stability. This feature is attributed to the various biomolecules involved in this process as they participate in overcoming the toxicity of the reagents, and contribute to the NP production and stabilization. One of the biomolecules most found adsorbed on the S-NP surface is L-cysteine, especially produced by bacteria and fungi [131,200,201]. In addition to its function as a capping agent, it is also the source of sulfur for S-NP formation through the catalytic activity of several enzymes, such as cysteine desulfhydrase, cysteine synthase, cystathionine γ-lyase, and threonine dehydratase [77,80,95,96,130,200,202]. Moreover, Dunleavy et al. showed that QD growth and stability depend also on the concentration of sulfur source and availability of capping agents [200]. For instance, if only L-cysteine is supplemented, sulfur production owing to the action of smCSE could not last more than 4 h therefore the obtained CdS NPs would precipitate if this period was exceeded. On the other hand, the addition of glutathione allowed the NP formation for longer times, especially at 10 mM concentration, reaching the maximum production after 24 h as sulfur was directly enzymatically extracted from L-cysteine whereas glutathione increased the capping agent availability, thus improving NP formation in terms of yield and stability. Furthermore, the addition of glutathione yielded the formation of smaller NPs due to its capping role [140]. This was recently corroborated by Ma et al. who highlighted the interesting effects induced by glutathione addition into the reaction medium containing *P. stutzeri* threonine dehydratase and L-cysteine [202]. As a result, smaller, well dispersed and more stable CdS NPs were obtained.

**Figure 5:**
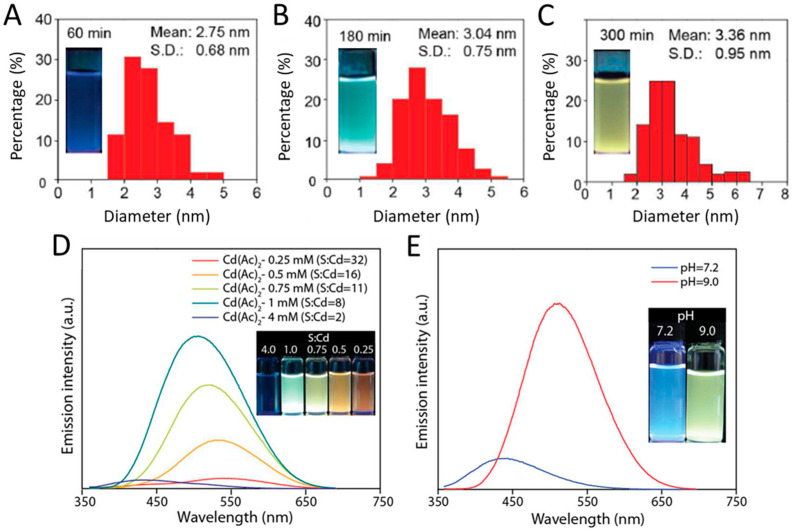
Impact of the experimental parameters on the size of CdS QDs synthesized using cadmium-tolerant *Stenotrophomonas maltophilia* in the presence of cadmium acetate and L-cysteine. (**A**–**C**): Impact of reaction time on the size. Insert: Pictures of the corresponding colloids. Adapted from Ref. [131] with permission from the Royal Society of Chemistry; (**D**) Impact of S:Cd ratio on CdS QD emission spectra. Insert: Picture of CdS colloids made at different S:Cd ratios; (**E**) Impact of pH on CdS QD emission spectra. Insert: Picture of CdS colloids made at pH 7 and 9. Adapted from Ref. [77] with permission from the American Chemical Society.

Many studies have demonstrated that the reaction time is an essential parameter in NP growth and size control. As a consequence of particle size variation, the optical properties evolve too; this can be easily monitored using fluorescence and ultraviolet-visible spectroscopy [41,103,177]. For instance, Gallardo et al. showed a size increase of CdS NPs synthesized using the cell-free extract of *P. fragi* GC01, 2 mM cysteine and 20 µg mL^−1^ CdCl_2_. The resulting fluorescent sample turned from green to yellow to eventually orange for 1, 2, and 3 h of reaction time, respectively, while their average diameter measured using TEM were 2.31 ± 0.51, 2.59 ± 0.71, and 2.59 ± 0.78 nm, respectively [80]. Similar results were obtained by Yang et al. who observed the redshift witnessed by both the CdS NP absorption and emission spectra as the pH increased and reaction time increased denoting the size increase of the NPs, as confirmed by TEM micrographs, from 2.75 to 3.36 nm for reaction times of 60 and 300 min, respectively (Figure 5) [77,131]. These findings were corroborated by Órdenes-Aenishanslins et al. who used *E. coli* to tune the size of spherical CdS QDs by controlling the reaction time [103].

Other experimental parameters, such as the pH, concentration and ratio of metal precursor and sulfur source impact the S-NP size and stability. For instance, changing the S:Cd ratio notably affects the NP size and optical properties [77]. The optimal ratio should be determined as cadmium concentrations impact the toxicity and the ability of the microorganism to efficiently perform the biosynthesis. On the other hand, L-cysteine affects the amounts of generated S^2−^ and capping agent availability. In addition to reaction time, biogenic CdS QDs are also strongly affected by the pH as this alters the appropriate physiological conditions of used microorganisms and affects the conformation of involved biomolecules [41,70,77].

## 4. Biomedical Applications

Nanomaterials have shown to be highly beneficial in biomedicine and are successfully used for biomedical applications. Some NPs have already been approved for clinical uses to treat, cure, or diagnose diseases, while many other NPS continue to emerge in research stages [211]. There are many fields in which NPs have shown promising results, such as, for example, tumor research [212,213], bioimaging [214,215], drug delivery [216,217], cancer photodynamic and photothermal therapy [218,219,220,221], and antimicrobial activity [84,222]. Among the biogenic S-NPs described here, QDs constitute the predominant class used for specific bio-applications. Besides being applied in optoelectronic devices, sensing, and photocatalysis [223,224,225,226,227], these NPs are gaining interest in the biomedical field [15,29,38].

**Figure 6:**
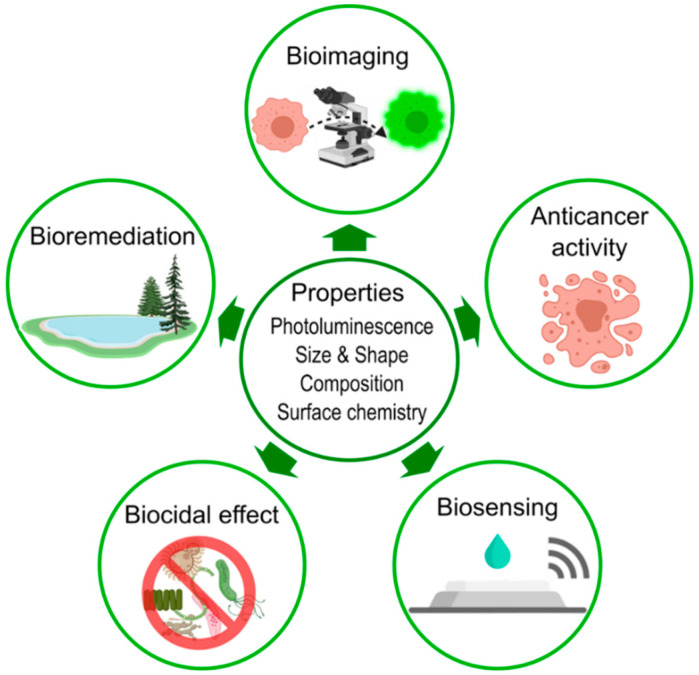
Properties and bio-applications of biogenic S-NPs.

To be applied in the biomedical field, NPs must fulfill some strict requirements [15,218]. Water solubility, or hydrophilicity, is essential since these materials will evolve and interact within aqueous systems in the body (animal or human) and disperse to target tissues. Surface chemistry, functional groups, and charge are fundamental to avoid being recognized by the immune system and eliminated. Additionally, functional groups directly affect the NP stability. Therefore, it is imperative to consider the nature of ligands and adsorbed moieties to confer the NPs, via surface modification techniques, the appropriate character, such as hydrophilicity, or amphiphilicity, to avoid their aggregation, achieve improved stability, and implement the adequate targeting and/or drug loading when needed [212,218]. The following paragraphs discuss some of the emerging bio-applications of biosynthesized S-NPs, especially in cancer research, bioimaging, biosensing, antimicrobial activity, bioremediation, and photocatalysis (Figure 6).

### 4.1. Cancer Treatment

NPs have driven an extraordinary revolution in the field of cancer research as they are used for the diagnostics, imaging, and therapy [228,229]. There are few studies reporting the use of biogenic S-NPs in this field. In the work of Shivaji et al., cytotoxicity tests were performed using the human lung alveolar basal epithelial cell line (A549) treated with the biosynthesized CdS QDs and cisplatin, a standard drug used to treat different types of cancer [19]. As a result, A549 treated with CdS QDs and cisplatin at concentrations of 10, 20, 30, 40, and 50 μg mL^−1^ for 24 h underwent a more significant cell inhibition at 20 μg mL^−1^ or higher concentrations, unlike when treated by cisplatin alone. This might be attributed to the fact that QDs could affect DNA replication and inhibit the enzymatic activity causing, thus, cell apoptosis. In a similar approach, Alsaggaf et al. determined the inhibitory concentration, IC_50_, of CdS NPs for the carcinogenic cell lines MCF7, PC3, and A549, to be 190, 246, and 149 μg mL^−1^, respectively. This inhibitory response is ascribed to the oxidative environment stemming from the release of metal ions by the biogenic CdS NPs [92].

### 4.2. Bioimaging, Biodetection and Biosensing

In the bioimaging field, QDs are preferred because of their superior fluorescent properties [15]. These NPs display a wide emission range of visible and infrared wavelengths depending on the size, shape, and composition [214]. The fluorescence of QDs can reach intensities more than ten times greater than that of traditional fluorescent dyes as well as being brighter and more photostable due to lower photobleaching compared to organic fluorophores [15,212]. In that sense, QDs have been extensively utilized for biomedical interests, principally in the bioimaging field [19,52,103].

Some reports highlight that biosynthesized NPs can be used to visibly detect specific organisms and plant cells. For instance, Qi et al. developed a method to detect specific bacteria by adding cadmium ions to cultures of *P. aeruginosa*, *E. coli*, *S. aureus*, *Vibrio alginolyticus*, and SRB [114]. Two days later, the recorded fluorescence was only visible in the SRB sample because of its ability to produce CdS NPs that tend to accumulate at their cell wall. Imaging plant tissues using bioproduced S-NPs is also possible. For example, Borovaya et al. imaged the *Allium cepa* (garlic) epidermal root cells using bioproduced Ag_2_S NPs since these cells emitted luminescence in the green region (520–550 nm) [182].

Moving forward, many reports have shown that biogenic S-NPs can be applied successfully for in vitro bioimaging of specific cells. To illustrate this, the human breast adenocarcinoma cell line (MCF-7) was imaged using CdS and CdTe QDs produced by *R. stolonifer* [174]. Similarly, CdSAg ternary QDs were formed using *E. coli* and used to label HeLa human cells, which provided fluorescence responses in the near-infrared region allowing their visualization without evoking morphology changes [103]. In a novel approach, photoluminescent CuInS_2_/ZnS core/shell QDs, biosynthesized utilizing the single enzyme cystathionine γ-lyase (CSE) and subsequently conjugated with IgG antibodies to specifically bind to epidermal growth factor receptor, were found to bind to specific sites of THP-1 leukemia cells after 1-h incubation, unlike their non-conjugated analogs for which no specific binding was seen using confocal microscopy [201]. Besides, the cell viability was monitored every 20 min for 6 h. Neither imaging process nor QDs caused adverse or toxic effects [201]. Lastly, the in vivo bioimaging of MDA-MB-231 breast cancer cell line in mice was performed using fluorescent CdS_x_Se_1−x_ QDs synthesized using *E. coli* [132].

### 4.3. Antimicrobial Activity

The agar-disk and the agar-well diffusion methods are standard antimicrobial tests performed using NPs [84,92,141]. Both methods evaluate cell cultures on agar plates [230]. In the first case, filter paper disks containing the test compound, NPs in the present case, at a certain concentration are placed on the agar surface whereas in the second, holes are punched in the agar and filled with the test compound [230]. The antimicrobial activity starts with the interaction between NPs and the cell wall of the microbes [84,88,182]. At this level, electrostatic interactions occur by the difference in electrical charges of the negatively charged cell membrane and NPs. This interaction induces conformational changes on the cell membrane, therefore affecting the cell permeability and other vital metabolism pathways [88,182]. It has been shown that NPs can enter inside the cells, producing adverse effects on the cell enzymatic machinery and on the genetic material [19,222]. Consequently, protein translation from DNA is irreversibly damaged, resulting in cell death [222]. Besides, the NPs can release, in a controlled manner, heavy cations to the medium which enter the cell, generating free radicals, like ROS, that induce the cell death [10,71,222].

NPs have been widely used as antimicrobial agents [222] and are particularly promising to replace antibiotics due to their potent biocidal activity [93,222]. Thus, the application of NPs could reduce the economic costs associated with cleaning, disinfection, and sterilization, and to antibiotic development and production. For instance, Rajeshkumar et al. performed antibacterial tests with the bioproduced CdS and ZnS NPs against four oral pathogens: *Streptococcus* sp., *Staphylococcus* sp., *Lactobacillus* sp., and *C. albicans* [88]. In general, CdS and ZnS NPs produced considerable zones of inhibition; these diameters varied between 10–25 mM depending on the NP concentration and considered pathogens [88]. As a hypothetical mechanism of action, the NPs are impregnated to the pathogen cell membrane and simultaneously discharge toxic ions to the cell that interrupt important metabolic proteins, thus inhibiting the cell growth [88]. Moreover, bioproduced ZnS NPs triggered *E. coli* cell death because of NP release of metal cations that interact with the cells and generate ROS [71].

Although NPs demonstrate antimicrobial activity, the detailed mechanisms are not completely understood. It appears that some organisms have better resistance than others while particular species remain unaffected. For instance, fungus-mediated biogenic CdS NPs inhibit the cell growth principally of Gram− and + bacteria while they do not affect the yeast *C. albicans* [92]. Besides, Borovaya et al. showed that biosynthesized Ag_2_S NPs had higher antibacterial activity against the Gram− *E. coli* (90%) than against the Gram+ *Bacillus thuringiensis* (70%) [182]. In another work, CdS NPs synthesized by a plant extract effectively inhibited the growth of both Gram− and + bacteria since these tiny NPs penetrate the bacterial cell wall indiscriminately to cause lethal effects [19].

### 4.4. Environmental Sensing and Bioremediation

Biogenic S-based NPs can sense heavy metal cations in aqueous solutions. For instance, CdS QDs synthesized using pomegranate peel extract selectively detected Pb^2+^ as the fluorescence redshifts in their presence at different concentrations [74]. It is believed that Pb^2+^ electrostatically interacts with the capping agents by aggregating at the NP surface and eliciting optical changes. Similarly, biogenic PbS NPs detect concentrations of As(III) as low as 50 ppb, even in the presence of other metal ions at reduced concentrations, since these cations most likely bound NP surface thiol groups stemming from cysteine [180]. Furthermore, biogenic S-based NPs have shown potency to remove contaminants from water. For instance, NPs of different shapes produced using *Clostridiaceae* sp. were successfully tested in the UV-mediated photocatalytic degradation of methylene blue [100]. Importantly, the cuboidal NPs exhibited the best catalytic activity by completely removing this dye within 20 h of UV light irradiation. The surface, size, and shape played essential roles in this study [100]. Similar results were also obtained using CdS NPs [89] as these NPs mediate the production of hydroxyl radicals from water that act as oxidizing agents converting MB into a harmless form. All these reports highlight the capabilities and potential bio-applications of biogenic S-based chalcogenide NPs for environmental purposes and pave the way to their future utilizations in water bioremediation. Additionally, the process of S-NP generation using biological entities may constitute by itself a valuable natural bioremediation process.

### 4.5. Other Applications

Besides the previous bio-applications, some studies tested the biogenic S-NPs in other fields. Owing to their electric properties, biogenic S-NPs have been used in solar cells [122,126] and diode fabrication [176]. For instance, CdS/CdSe core/shell QDs synthesized employing *E. coli* were tested in solar cells as photosensitizer materials and compared with other QDs. Core/shell QDs with an average size of 16.7 nm demonstrated improved photovoltaic parameters compared to CdS, and CdTe QDs produced by biological and chemical routes, respectively [122]. Some studies suggest that core/shell NPs produce better results than simple S-based NPs as photosensitizers in solar cells [76,122,126]. Although these NPs worked effectively, the efficiencies are not comparable to materials currently used in the electronic field.

## 5. Conclusions and Future Perspectives

This review provides an extensive update on the biological synthesis of various sulfide-based chalcogenide NPs, such as CdS, PbS, and Ag_2_S, using various biological resources, namely bacteria, yeast, fungi, algae, plants, viruses and identified biomolecules. Different methods of S-NPs biogenesis are discussed as well as the factors that impact the properties of the resulting S-NPs. Among these nano-objects, CdS NPs remain, by far, the most reported biogenic nanomaterials in the literature. Depending on the bioresource used, the S-NP biosynthesis might be intra- or extra-cellular. The examples examined here show that yeast produces S-NPs via an intracellular process. On the other hand, fungi promote S-NP formation via an extracellular pathway. In the case of bacteria, there is no privileged pathway as some findings demonstrated that the biosynthesis of S-NPs is intracellular whereas others found it extracellular. Besides, some bacteria are able to carry out this process in both ways. Generally, viruses synthesize first the S-NPs and then serve as a template for the growth of nanotubes owing to the merging of individual S-NPs. Interestingly, some experimental protocols require the presence of an external sulfur source for the formation of S-NPs to take place while others rely on S-containing biomolecules made available by the used bioresource. Screening the experimental parameters, such as the bio-entity, precursor concentration and nature, presence of additional reagents, reaction time, and pH, enables the formation of S-NPs with desired features in terms of composition, size, shape, and colloidal stability. For instance, the bioproduced S-NPs display a variety of shapes, like needles, sheets, cubes, tubes, rods although spheres remain dominant. In addition to solar cells and electronics, some of the as-produced biogenic S-NPs have proven efficient in several bio-applications, such as cancer therapy, bioimaging, biocidal activity and bioremediation.

Research is still needed to address key requirements that would make this green route for the synthesis of S-NPs an efficient alternative to its chemical analog. In this regard, future research should be directed to: (i) thoroughly understand the mechanisms that rule the S-NP biosynthesis; (ii) accurately identify the key molecules that are involved in their synthesis and impact their characteristics, such as the size, shape, and stability; (iii) explore the scalability of these processes; and (iv) confer these NPs specific surface functionalities by conjugating them using specific molecules and ligands to achieve a greater control on their characteristics, improve their biocompatibility, overcome their limitations and expand their bio-applications.

## Figures and Tables

**Table 1:** Biosynthesis of cadmium-based S-NPs using bacteria.

Type	Species	Mechanism	Added Sulfur Source ^a^	Shape	Size (nm)	Application	Ref.
CdS	*Acidithiobacillus* spp.	Int., Ext.	Cysteine and glutathione	(QDs)	~6, 10	-	[70]
	*A. thiooxidans* ATCC	Int., Ext.	-	(QDs)	~6.9, 10	-	[111]
	*Bacillus amyloliquifaciens*	Ext.	Na_2_S	Spherical	~3.2	-	[112]
	*B. licheniformis*	Ext.	Na_2_S	-	2–10	-	[102]
	*B. licheniformis*	Int.	Na_2_S	Spherical	~5.1	-	[93]
	*Citrobacter braakii*	Int.	Na_2_S	Spherical	50–100	-	[113]
	*Clostridium thermoaceticum*	Ext	-	Spherical	50	-	[96]
	*Desulfovibrio caledoiensis*	Int., Ext.	-	Spherical	40–50	Bioimaging	[114]
	*D. alaskensis*	Int., Ext.	-	-	10–46	-	[115]
	*D. desulfuricans*	Ext.	-	Spherical	40–80	-	[116]
	*Enterococcus* sp.	Ext.	CdSO_4_	Spherical	50–180	Antimicrobial	[88]
	*Escherichia coli*	Int.	-	Spherical, elliptical	2–5	-	[117]
	*E. coli*	Int.	-	Spherical	~10	-	[118]
	*E. coli*	Int.	L-cysteine, glutathione, mercaptosuccinic acid	-	7.5, 3.5	-	[119]
	*E. coli* *	Int.	Cysteine	-	2–5	-	[120]
	*E. coli* *	Int.	Na_2_S	-	~6	-	[121]
	*E. coli*	Ext.	Cysteine	Spherical	3–9	-	[103]
	*E. coli*	Ext.	L-cysteine	Spherical	~12	Solar cells	[122]
	*E. coli*	-	Na_2_S	Triangular	40–80	Antimicrobial	[123]
				Irregular	40–80		
	*E. coli* JM109 *	Int.	Na_2_S	-	2–6	-	[124]
	*E. coli* R189 *	Int.	Na_2_S	-	3–4	-	[124]
	*Halobacillus* sp.	Ext.	Cysteine	Hexagonal	~4	-	[41]
	*Klebsiella pneumoniae*	Ext.	CdSO_4_	Spherical	10–25	Antimicrobial	[84]
	*K. pneumoniae*	Ext.	Growth medium (FeSO_4_, MgSO_4_, (NH_4_)_2_SO_4_)	Spherical	5–200	-	[83,97]
	*Lactobacillus* sp.	Ext.	H_2_S ^b^	Spherical	3.5–5.5	-	[125]
	*Pedobacter* sp.	Ext.	-	-	~2.8, 4.9	Solar cells	[126]
	*Pseudomonas aeruginosa*	-	-	Spherical	10	-	[127]
	*P. putida*	Int.	Cysteine	-	12.5–27.5	-	[128]
	*Pseudomonas* spp.	Int.	-	-	-	-	[129]
	*Pseudomonas* spp.	Int.	-		10–40	-	[130]
	*Pseudomonas* spp.	Int., Ext.	-	Cubic	2–16	-	[80]
	*Psychrobacter* spp.	Int.	-	-	-	-	[129]
	*Rhodobacter sphaeroides*	Int.	Growth medium (MgSO_4_, (NH_4_)_2_SO_4_)	Spherical	~2.3, 6.8, 36.8	-	[79]
	*Rhodopseudomonas palustris*	Int.	CdSO_4_	Spherical	~8	-	[85]
	*Shewanella* sp.	Int.	-	-	-	-	[84]
	*Stenotrophomonas maltophilia*	Ext.	L-cysteine	Spherical	~2.75, 3.04, 3.36	-	[131]
	*S. maltophilia*	Ext.	L-cysteine	Spherical	~4.3, 4.8	-	[77]
CdSAg	*E. coli*	Ext.	Cysteine	Spherical	6–9	Bioimaging, solar cells	[103]
CdS/ CdSe	*E. coli*	Ext.	L-cysteine	Spherical (core/shell)	~17	Solar cells	[122]
CdS_x_Se_1−x_	*E. coli*	Int.	-	Spherical	2.0 ± 0.4	Bioimaging	[132]

^a^: If no external source is added or mentioned, the sulfur source might be the culture media and/or biomolecules; ^b^: Knowing that the boiling point of H_2_S is −60 °C, Na_2_S or a similar product was most likely used instead. Int.: Intracellular; Ext.: Extracellular; ~: Approximately; *: Genetically engineered strain.

**Table 2:** Biosynthesis of zinc-, lead-, silver-, arsenic-, bismuth- and copper-based S-NPs using bacteria.

Type	Species	Mechanism	Added Sulfur Source ^a^	Shape	Size (nm)	Application	Ref.
ZnS	*Clostridiaceae* sp.	Ext.	Na_2_SO_4_ andZnSO_4_	Spherical	5.95–3.34	-	[140]
	*Desulfobacteriaceae* sp.	Int.	SO_4_	Spherical	2–5	-	[138]
	*Desulfovibrio desulfuricans*	Ext.	ZnSO_4_	-	~2.4	-	[87]
	*D. desulfuricans*	-	Na_2_S	Amorphous	4–12	-	[144]
	*D. desulfuricans*	Ext.	ZnSO_4_	Spherical	20–30	-	[145]
	*Klebsiella pneumoniae*	Ext.	ZnSO_4_	Spherical	65	Antimicrobial	[84]
	Mix of SRB	Ext.	-	-	~6.5	-	[104]
	Mix of SRB	Ext.	Na_2_SO_4_	Spherical	15	-	[142]
	*Rhodobacter sphaeroides*	Ext.	ZnSO_4_	Spherical	~4, 8, 30, 105	-	[146]
	*Serratia nematodiphila*	Ext.	ZnSO_4_	Spherical	80	Antimicrobial	[141]
	SRB from peatlands	Ext.	-		12–14	-	[139]
	*Thermoanaerobacter* sp.	Ext.	-	Spherical	2–10	bio-ink	[147]
	*Thermoanaerobacter* sp.	Ext.	-	Spherical	~2	-	[148]
γ-MnS	*Clostridiaceae* sp.	Ext.	Na_2_SO_4_	Hexagonal	2–3 μm D., 200–300 nm T. ^‡^		[72]
PbS	*Clostridiaceae* sp.	Ext.	Growth medium (MgSO_4_, Na_2_SO_4_)	Nanocuboids	50 × 50 × 100	As(III) detection	[100]
				Nanosheets	10		
				Nanospheres	60		
	*Desulfotomaculum* sp.	Ext.	Growth medium (MgSO_4_, Na_2_SO_4_)	Spherical, nanorods	13	-	[137]
	*Rhodobacter sphaeroides*	Ext.	Growth medium (MgSO_4_, (NH_4_)_2_SO_4_)	Spherical	~10.5	-	[149]
	*Shinella zoogloeoides*	Ext.	-	-	-	-	[150]
	*Stenotrophomonas maltophilia*	Ext.	L-cysteine	Amorphous	~3	Solar cells	[76]
PbS/CdS	*S. maltophilia*	Ext.	L-cysteine	Amorphous	4–5	Solar cells	[76]
Ag_2_S	*Escherichia coli*	Ext.	L-cysteine	Spherical	<15	-	[103]
	*Pseudomonas stutzeri*	Int.	-	Triangular, hexagonal	up to 200 nm	-	[151]
	*Shewanella oneidensis*	Ext.	Na_2_S_2_O_3_	Spherical	~9 ± 3.5	-	[152]
	*S. oneidensis*	Ext.	Na_2_S_2_O_3_	Spherical	2–16	-	[153]
As_-_S	*Desulfotomaculum auripigmentum*	Int., Ext.	Cysteine or SO_4_^2−^	Spherical	50–100	-	[154]
	*Shewanella* sp.	Ext.	S_2_O_3_^2−^	Nanotubes	20–100	-	[155]
	*Shewanella* sp.	Ext.	S_2_O_3_^2−^	Nanotubes	30–70	-	[156]
Bi_2_S_3_	*Clostridiaceae* sp.	Ext.	Bi_2_(SO_4_)_3_ and Na_2_SO_4_	Nanorods	100 nm D., 1000 nm L.	-	[157]
				Nanoneedles	10–20 nm D., 5–10 nm L.	-	
	*Clostridium acetobutylicum*	Ext.	Bi_2_(SO_4_)_3_	Hexagonal	6–10 or 440–500 ^‡^	-	[158]
CuS	*S. oneidensis*	Ext.	CuSO_4_	Nanorods	17.4 nm D., 80.8 nm L.	-	[159]

^a^: If no external source is added or mentioned, the sulfur source might be the culture media and/or biomolecules. Int.: Intracellular; Ext.: Extracellular; ~: Approximately. ^‡^: These are microparticles according to the definition of nanomaterials adopted in the present article. D.: Diameter; L.: Length; T. Thickness.

**Table 3:** Biosynthesis of S-NPs mediated using fungi.

NP	Species	Mechanism	Added Sulfur Source ^a^	Shape	Size (nm)	Application	Ref.
CdS	*Aspergillus niger*	Ext.	Na_2_S	Spherical	2.7–7.5	Anticancer, antibacterial	[92]
	*Candida glabrata*	Int., Ext.	-	Spherical	~2		[108]
	*C. glabrata*	Int., Ext.	-	Spherical	~2		[109]
	*C. glabrata*	Int., Ext.	-	Spherical	~2	-	[110]
	*C. glabrata*	Int., Ext.	-	Spherical	~2	-	[105]
	*C. glabrata*	Int.	-	-	-	-	[170]
	*Coriolus versicolor*	Ext.	MgSO_4_	Spherical	8–15	-	[167]
	*Fusarium oxysporum*	Ext.	CdSO_4_	Spherical	5–20	-	[165]
	*Fusarium* sp.	Ext.	CdSO_4_	Spherical	80–120	-	[171]
	*Phanerochaete chrysosporium*	Ext.	Thioacetamide and mercaptoacetic acid	Spherical	1.5–2.0	-	[172]
	*Pleurotus ostreatus*	Ext.	CdSO_4_ and Na_2_S	Spherical	4–5	-	[173]
	*Rhizopus stolonifer*	Ext.	-	Spherical	~8.8	Bioimaging	[174]
	*Saccharomyces cerevisiae*	Ext.	H_2_S ^b^	Spherical	2.5–4.5	-	[125]
	*S. cerevisiae*	Ext.	Na_2_S	Spherical	~2	Solar cells	[168]
	*Schizosaccharomyces pombe*	Ext.	CdSO_4_	Spherical	2–6	-	[175]
	*S. pombe*	Int., Ext.	-	Spherical	~2	-	[108,110]
	*S. pombe*	Int.	CdSO_4_	-	2–2.5	Electronics	[176]
	*S. pombe*	Int.	-	-	-	-	[170]
	*Trametes versicolor*	Ext.	Thioacetamide and mercaptoacetic acid	Spherical	~6	-	[177]
	*Trichoderma harzianum*	Ext.	Na_2_S	Spherical	3–8	-	[169]
ZnS	*A. flavus*	Ext.	ZnSO_4_	Spherical	12–24	-	[71]
	*F. oxysporum*	Ext.	ZnSO_4_	Spherical	42	-	[166]
	*S. cerevisiae*	Int.	ZnSO_4_	Spherical	30–40	-	[178]
PbS	*Rhodosporidium diobovatum*	Int.	-	-	2–5	-	[179]
	*A. flavus*	-	Na_2_S	Spherical	35–100	As detection	[180]
	*Torulopsis* sp.	Int.	-	Spherical	5	-	[181]
Ag_2_S	*P. ostreatus*	Ext.	Na_2_S	Spherical	10–15	Antibacterial, bioimaging	[182]
CuS	*F. oxysporum*	Ext.	CuSO_4_	Spherical	2–5	-	[81,183]

^a^: If no external source is added or mentioned, the sulfur source might be the culture media and/or biomolecules; ^b^: Knowing that the boiling point of H_2_S is −60 °C, Na_2_S or a similar reagent was most likely used instead. Int.: Intracellular; Ext.: Extracellular; ~: Approximately.
